# Genome-Wide Screen of the Hippocampus in Aged Rats Identifies Mitochondria, Metabolism and Aging Processes Implicated in Sevoflurane Anesthesia

**DOI:** 10.3389/fnagi.2020.00122

**Published:** 2020-05-07

**Authors:** Yujie Wang, Min Qian, Yinyin Qu, Ning Yang, Bing Mu, Kaixi Liu, Jing Yang, Yang Zhou, Cheng Ni, Jing Zhong, Xiangyang Guo

**Affiliations:** ^1^Department of Anesthesiology, Peking University Third Hospital, Beijing, China; ^2^Department of Anesthesiology, National Cancer Center/National Clinical Research Center for Cancer/Cancer Hospital, Chinese Academy of Medical Sciences and Peking Union Medical College, Beijing, China; ^3^Department of Anesthesiology, Zhongshan Hospital, Fudan University, Shanghai, China

**Keywords:** genome-wide screen, anesthesia, mitochondria, metabolism, aging

## Abstract

Previous studies have shown multiple mechanisms and pathophysiological changes after anesthesia, and genome-wide studies have been implemented in the studies of brain aging and neurodegenerative diseases. However, the genome-wide gene expression patterns and modulation networks after general anesthesia remains to be elucidated. Therefore, whole transcriptome microarray analysis was used to explore the coding gene expression patterns in the hippocampus of aged rats after sevoflurane anesthesia. Six hundred and thirty one upregulated and 183 downregulated genes were screened out, then 44 enriched terms of biological process, 16 of molecular function and 18 of the cellular components were identified by Gene Ontology (GO) and KEGG analysis. Among them, oxidative stress, metabolism, aging, and neurodegeneration were the most enriched biological processes and changed functions. Thus, involved genes of these processes were selected for qPCR verification and a good consistency was confirmed. The potential signaling pathways were further constructed including mitochondrion and oxidative stress-related *Hifs*-*Prkcd*-*Akt*-*Nfe2l2*-*Sod1* signaling, multiple metabolism signaling (*Scd2*, *Scap*-*Hmgcs2*, *Aldh18a1*-*Glul* and *Igf1r*), as well as aging and neurodegeneration related signaling (*Spidr*-*Ercc4*-*Cdkn1a*-*Pmaip1* and *Map1lc3b*). These results provide potential therapeutic gene targets for brain function modulation and memory formation process after inhaled anesthesia in the elderly, which could be valuable for preventing postoperative brain disorders and diseases, such as perioperative neurocognitive disorders (PND), from the genetic level in the future.

## Introduction

Neurodegenerations, including Alzheimer’s disease (AD), represent important causes for the brain’s aging processes and related cognitive dysfunction and dementia (Wyss-Coray, 2016). For the pathogenesis of stroke, conventional risk factors explain only a small proportion of causes, and evidence from twins and family history study suggests that genetic predisposition is important (Dichgans, 2007). In common with many other complex diseases, in which environmental risk factors are thought to interact with multiple genes, the identification of the underlying molecular mechanisms and genes contributing to degenerated brain diseases is valuable and challenging. Candidate gene studies have produced few replicable associations (Dichgans and Markus, 2005). More recently, genome-wide association studies, using microarray platforms, have allowed a deeper understanding of the molecular factors involved in the pathophysiology of degenerated brain disease. These studies identified multiple susceptibility loci for neurodegeneration (Harold et al., 2009; Lambert et al., 2009; Seshadri et al., 2010; Hollingworth et al., 2011), and these genes were clustered into pathways including inflammation and immune response, lipid metabolism, endocytosis/intracellular trafficking (Kunkle et al., 2019). Studies also found that oxidative stress is associated with neurodegenerative disorders (Coyle and Puttfarcken, 1993). And oxidative stress (Chamorro et al., 2016), lipid metabolism, blood circulation (Ji et al., 2017), multi-organism process, protein catabolic metabolism (Cui et al., 2018), and autophagy (Menzies et al., 2015) are the major shared genetic etiologies for stroke. Nevertheless, the are few studies about the gene network and pathophysiology for brain function modulation during the perioperative context.

Sixty-six million patients over 65 years of old worldwide undergo surgeries each year, including 8.5 million AD patients (Xie and Xu, 2013). Up to 40% of these patients suffer from perioperative neurocognitive disorders (PND), which includes postoperative cognitive dysfunction, postoperative delirium, et cetera (Monk et al., 2008; Evered et al., 2018). Anesthesia, surgical trauma, aging, as well as preoperative cognitive impairment propose to the onset of PND (Monk et al., 2008; Schenning et al., 2016; Racine et al., 2018). Meanwhile, neuroinflammation, mitochondrial dysfunction and oxidative stress (Fischer and Maier, 2015), DNA damage and apoptosis (Madabhushi et al., 2014), synaptic plasticity dysfunction (Li X. M. et al., 2014), amyloid plaques and neurofibrillary tangles (Ni et al., 2013) could be the contributing pathological factors. Ours and related researches have indicated that inhaled general anesthesia plays a major role in the PND (Xu et al., 2014; Ni et al., 2015), however, the gene expression patterns and modulation networks during general anesthesia remains to be elucidated. Therefore, we used the genome-wide screen to explore the gene expression patterns in the hippocampus of aged rats after sevoflurane anesthesia. And we established functional annotation of differentially expressed genes, modulation networks, as well as potential signaling pathways during the process, to provide insights into the monolithic mechanisms for inhaled anesthesia and related brain function modulation and memory formation.

## Materials and Methods

### Animals

Male Sprague–Dawley rats, 18-month old, weighing 550–600 g, were used in the studies. Before sevoflurane exposure, the rats were maintained on a standard housing condition with food and water *ad libitum* for 2 weeks.

### Rat Anesthesia

The animal protocol was approved by the Peking University biomedical ethics committee experimental animal ethics branch (No. LA2018085). The rats were randomly assigned to control and sevoflurane groups. Minimum alveolar concentration (MAC) of sevoflurane for aged rats has been reported as 2.4–2.7% (Li X. Q. et al., 2014). In the present study, rats in the sevoflurane group received 2.5% sevoflurane in 100% oxygen for 4 h in the anesthetizing chamber, whereas the control group received 100% oxygen for 4 h in an identical chamber. The rats breathed spontaneously, and the anesthetic and oxygen concentrations were monitored continuously (Datex, Tewksbury, MA, USA). Temperature of the anesthetizing chamber was controlled to maintain the rectal temperature of the animals at 37 ± 0.5°C. Four hours sevoflurane anesthesia has been shown not to significantly alter values of blood pressure and blood gas in our preliminary experiment. After the termination of sevoflurane anesthesia, rats were placed in a chamber containing 100% oxygen until the regain of consciousness 20 min later. The rats were sacrificed by decapitation at the end of the experiments. The brain tissues were removed, and the hippocampus was dissected out and frozen in liquid nitrogen for the subsequent experiments.

### RNA Extraction and Quantification

Total RNAs were isolated from the hippocampus using trizol reagent (Invitrogen, Carlsbad, CA, USA), then digested with RNase-Free DNase to remove residual DNAs. The RNA concentrations were analyzed using the Nanodrop2000 (Thermo Fisher Scientific), then total RNA (2 μg) was reverse-transcribed using the GoScriptTM ReverseTranscription System (Promega, Madison, WI, USA).

### Affymetrix Whole Transcriptome Microarray Analysis and Functional Annotation

Whole transcriptome microarray analysis was performed using GeneChip™ Rat Transcriptome Array 1.0 (Affymetrix, Santa Clara, CA, USA), and the result data were deposited in NCBI with the GEO accession code GSE141242. Briefly, isolated RNA (100 ng) was mixed with 1.5 μl of Poly-A RNA control solution and subjected to reverse transcription. The obtained cDNA was used for *in vitro* transcription to prepare antisense RNA (aRNA) by incubation at 40°C for 16 h. Then, the aRNA was applied for the second round of sense cDNA synthesis using the WT Expression kit (Ambion, Austin, TX, USA). The obtained cDNA was used for biotin labeling and fragmentation by Affymetrix GeneChip^®^ WT Terminal Labeling and Hybridization. Biotin-labeled fragments of cDNA (5.5 μg) were hybridized to the Affymetrix^®^ Rat Transcriptome Array Strip (45°C/24 h), and up to 25 unique probe sequences were hybridized to a single transcript. Following hybridization, each array strip was washed and stained using the Fluidics Station of GeneChip^®^ Scanner 3000 7G system (Affymetrix, Santa Clara, CA, USA). The array strips were scanned using the Imaging Station of the GeneChip^®^ Scanner 3000 7G system. Gene Ontology (GO) functional annotation and Kyoto Encyclopedia of Genes and Genomes (KEGG) pathway enrichment analyses were performed for DEGs using Database for Annotation, Visualization, and Integrated Discovery (DAVID^1^). GO enrichment analysis contains three categories: biological process, molecular function, and cellular component.

### Quantitative Real-Time PCR (qPCR)

The significances of genes changes were calculated by −log10 (*p*-value), and higher −log10 (*p*-value) indicated that the gene was with more significant changes. We selected the top differentially expressed genes for qPCR verification.

qPCR was performed on the CFX96 Real-Time PCR Detection System (Bio-Rad, Hercules, CA, USA). Amplification mixture consisted of PowerUpTM SYBR^®^ Green master mix (Thermo Fisher Scientific), 10 μM forward and reverse primers (Invitrogen, Carlsbad, CA, USA) and approximately 1.5 μl of cDNA template. Primer sequences were obtained from the literature and checked for their specificity through in silico PCR. The forward and reverse primers are shown in Table 1. Amplification was carried out with an initial denaturation step at 95°C for 2 min followed by 45 cycles of 95°C for 10 s, 55°C for 30 s and 60°C for 30 s, then 65°C for 2 min in 10 μl reaction volume. All reactions were run in duplicate and the results were averaged from six independent studies. qPCR was quantified in two steps, first, β-actin levels were used to normalize target gene levels [ΔCycle threshold (ΔCt) = Cttarget gene—Ctβ-actin, target gene level = 2-ΔCt]. Second, the target gene levels of the sevoflurane group were presented as the percentage of those of the control group, and 100% of the target gene levels referred to the control levels.

**Table 1:** The forward and reverse primers for qPCR.

Genes	Primers	Sequence (5′ to 3′)
*Hif3a*	Forward primer	CACATGGACTGGGACCAAGACAGG
	Reverse primer	GTGTAGGCTGCTGGTGTGGAGTGT
*Epas1/Hif2a*	Forward primer	TCACTCATCCTTGCGACCAC
	Reverse primer	CAGGTGGCCGACTTAAGGTT
*Akt*	Forward primer	CACAGGTCGCTACTATGC
	Reverse primer	GAGACAGGTGGAAGAAGAG
*Nfe212*	Forward primer	GCACATCCAGACAGACACCA
	Reverse primer	GGCTGGGAATATCCAGGGCA
*Prkcd*	Forward primer	CCATCTCATCTGTACCTTCC
	Reverse primer	CCATCCTTGTCCAGCATTA
*Sod1*	Forward primer	TGACTTGGGCAAAGGTGGAA
	Reverse primer	ACAGTTTAGCAGGACAGCAGA
*Scap*	Forward primer	TATCTGGTGGTGGTTATTGG
	Reverse primer	GCATCTGGAGGAAGAAGTC
*Aldh18a1*	Forward primer	GTCCAGGAAGCCATTGAT
	Reverse primer	GCAACCACTTAGTAGTTAGC
*Glu1*	Forward primer	GCTGCCACACCAACTTTAGC
	Reverse primer	CAATCCGGGGAATGCGGATA
*Hmgcs2*	Forward primer	AGGAGGCCAATCCATACAACCA
	Reverse primer	TGGGGAAGGTCTGTATCCCT
*Igf1r*	Forward primer	GAACCGCATCATCATAACG
	Reverse primer	CACAGCCTTGACATAGACT
*Scd2*	Forward primer	CATCATCGCCAACACCAT
	Reverse primer	GGCTTGTAATACCTCCTCTG
*Spidr*	Forward primer	AGAAAGCTACAAGAGGAAGGACA
	Reverse primer	GGCATAGAAGCTGCAACGTG
*Pmaip1*	Forward primer	CACGATGAGAAGAAGCCCAA
	Reverse primer	AGTTTCTGCCGTAAATTCACT
*Ercc4*	Forward primer	AAGACAATCCGCCATTACT
	Reverse primer	ACGAGCATTCACGAACAT
*Map1lc3b*	Forward primer	GTGATTATAGAGCGATACAAGG
	Reverse primer	GGAGGCATAGACCATGTAC
*Cdkn1a*	Forward primer	ACCAGCCACAGGCACCAT
	Reverse primer	CGGCATACTTTGCTCCTGTGT
*Hipk2*	Forward primer	ACAGACTCACCGTATCCTT
	Reverse primer	TCCTCTTATCAGCATCTATGG
*Mal*	Forward primer	TGTCTGTGTTCTGCTTCC
	Reverse primer	TCTCATGGTAATGCCTGTAG
*Bmpr1b*	Forward primer	CCCAATCGATGGAGCAGTGA
	Reverse primer	ACGTTCAAGGCTTGGGCTAA
*β-actin*	Forward primer	AGAGCTATGAGCTGCCTGA
	Reverse primer	AATTGAATGTAGTTTCATGGATG

### Immunofluorescence Analysis

Immunofluorescence was performed to determine the expression of typical genes, as described in our previous studies (Ni et al., 2015). The hippocampus was fixed with 4% paraformaldehyde for 24 h, cryoprotected with 30% sucrose for 48 h, and sectioned using a cryostat (Cryotome E, Thermo Fisher, Waltham, MA, USA). Coronal sections (10 μm thickness) were incubated with HIF3α antibody (1:200 dilution; Abcam, Cambridge, UK), HMGCS2 antibody (1:100 dilution; Abcam, Cambridge, UK) or p21 antibody (1:100 dilution; Abcam, Cambridge, UK) overnight at 4°C, followed by incubation with a goat anti-rabbit FITC conjugated antibody (1:400 dilution; Servicebio, Wuhan, China) for 50 min at room temperature. Nuclei were subsequently counterstained with DAPI (1:5,000 dilution; Servicebio, Wuhan, China) for 10 min at room temperature. Images were captured using a Nikon Eclipse Ti confocal microscope. Hippocampal subregions CA1 and DG serve important roles in memory formation and other functions, these two regions were analyzed for HIF3α, HMGCS2, and p21 expressions.

### Fear Conditioning Test (FCT)

The FCT (Xeye CPP, Beijing MacroAmbition S&T Development, Beijing, China) was used to assess the cognitive function of rats after sevoflurane anesthesia as described in previous studies (Dong and Li, 2014; Cheng et al., 2015) with modification. FCT consisted of a training process at 3 h after the sevoflurane anesthesia and the evaluations at 2 and 7 days after anesthesia. In the training process, rats were placed in the context chamber to acclimate for 180 s, then they received a 2 Hz pulsating tone (80 dB, 3,600 Hz) for 60 s co-terminated with a mild foot shock (0.8 mA, a 0.5 s). In the evaluations, the hippocampal-dependent memory was assessed by the freezing time during exposure to a novel context test (the test was performed in the same chamber but with no cues or shock), while the hippocampal independent memory was assessed by the freezing time during exposure to the tone stimulus (the test was performed in an alternative context and with no shock; Chowdhury et al., 2005).

### Statistical Analysis

Statistical analysis was performed with Graphpad Prism 7.0 software. Quantitative data are presented as the mean ± SD. Non-paired two-tailed Student’s *t*-test was used to determine significant differences between the two groups. One-way ANOVA with Bonferroni’s multiple comparison test was used to analyze significant differences between multiple groups. *p* < 0.05 was considered significant. The microarray analysis was performed by Expression Console and Transcriptome Analysis Console Software. One-way ANOVA was applied. The *p*-value was adjusted with the FDR method (Benjamini Hochberg procedure). RNAs were screened with *p* < 0.05. The significance of GO and KEGG enrichment was calculated by the hypergeometric distribution and Fisher exact test, and a lower *p*-value indicated that the specific term was more significantly enriched. Two-way repeated-measures analysis of variance followed by a *post hoc* Bonferroni test was performed to analyze the results of behavioral studies. Values of *p* < 0.05 were considered to be significant.

## Results

Aged rats were assigned to control and sevoflurane groups. The vital signs and arterial blood gas analysis results during anesthesia were within the normal range. Due to previous studies from ours and other groups, multiple pathophysiological changes in the hippocampus emerged 3–12 h after anesthesia, and for oxidative stress, even immediately after anesthesia (Zhang et al., 2012; Li et al., 2013). And inhaled anesthetics could affect the hippocampus related behavioral function from 3 h after anesthesia (Zhang et al., 2012), so the hippocampus was dissected and tested 3 h after anesthesia in the present study.

**Figure 1:**
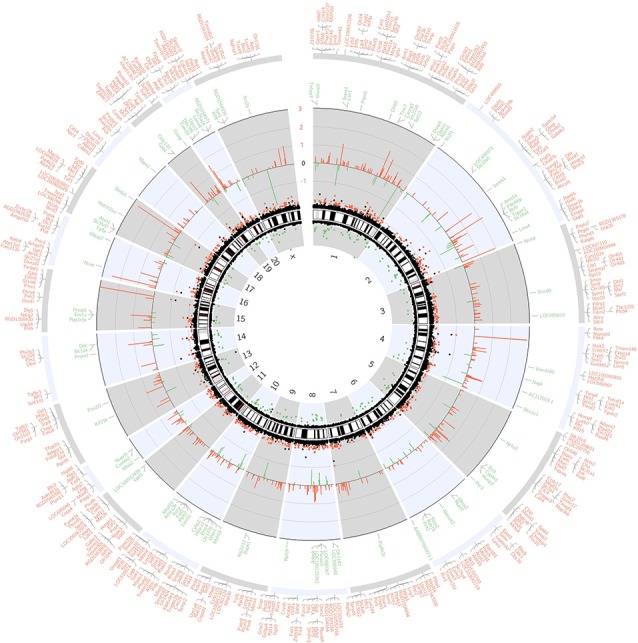
Circos plot of genome-wide coding gene expression differences of rat hippocampi in sevoflurane group vs. control condition (*n* = 3). The black innermost ring (with vertical lines) represents autosome ideograms (annotated is the chromosomal number), with the pter-qter orientation in a clockwise direction. Small red lines represent the centromeres within each chromosome. Dots outside the ideograms mark gene expression increase (red dots denote significantly increased mRNA signal), while dots inside mark gene expression decrease (green dots denote significantly decreased mRNA signal). The dot position marks the location of the Illumina 450K probe distribution along the genome. The second outermost black circle represents baseline (zero) and the β-value difference between isoflurane anesthesia and control condition. Red lines signify increased gene expression regions and green lines signify decreased gene expression regions, with the length of each line representing the difference level (*p* < 0.05). The last two circles show the RefSeq genes associated with different signal intensity (*p* < 0.05, and within the Ensembl database). Outer circle (red) shows genes with increased signal, and inner circle (green) shows genes with decreased signal.

The whole transcriptome gene expressions in the hippocampus of aged rats were detected by whole transcriptome microarray analysis (GeneChip™ Rat Transcriptome Array, *n* = 3). The microarray analysis was performed by Expression Console and Transcriptome Analysis Console Software. One-way ANOVA was applied and the *p*-value was adjusted with the FDR method. And the genome-wide map of all autosomal and heterosomal coding and complex genes was represented as a circular ideogram, composed of concentric circles depicting the entire autosome complement, with chromosomal location annotated in a clockwise manner and statistical significance indicated by radial arrangements and color codes. The black innermost ring (with vertical lines) represents autosome ideograms (annotated is the chromosomal number), with the pter-qter orientation in a clockwise direction. Small red lines represent the centromeres within each chromosome. Red dots outside the ideograms mark genes with expression (*p* < 0.05), while green dots inside mark genes with decreased expression (*p* < 0.05). The dot position marks the location of the Illumina 450K probe distribution along the genome. The second innermost black circle represents baseline (zero) and the β-value difference between sevoflurane and control groups. Red lines signify increased gene expression regions and green lines signify decreased gene expression regions, with the length of each line representing the difference level (fold change). The names of DEGs match the Ensembl gene database were listed in the two outermost circles, the first outermost circle listed DEGs with increased expression (Red), and the second outermost circle listed DEGs with decreased expression (green, Figure 1).

**Figure 2:**
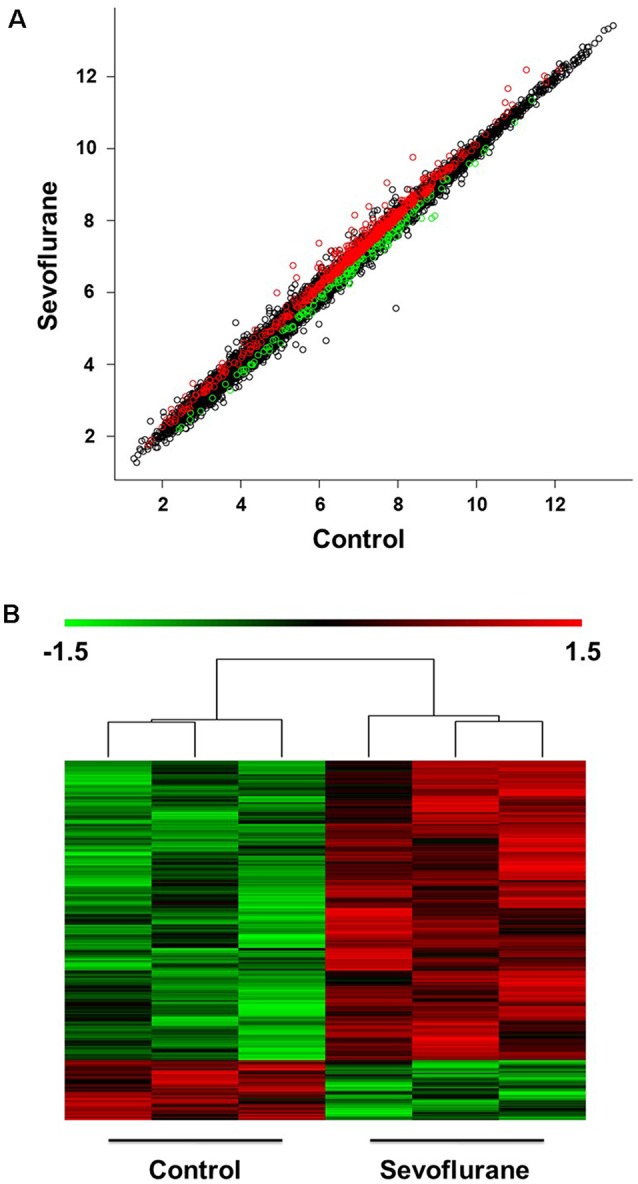
Differential expression of mRNAs in the hippocampus (*n* = 6). **(A)** The scatter plot indicated the variation in hippocampal gene expressions between the sevoflurane group and control condition. The values corresponding to the X- and Y-axes in the scatter plot are the normalized signal values of the control and sevoflurane groups (log_2_ scaled). **(B)** Hierarchical cluster analysis showed differentially expressed genes between groups from microarray analysis (*p* < 0.05).

The scatter plot indicated the variation in hippocampal gene expressions between the sevoflurane group and control condition. The values corresponding to the X- and Y-axes in the scatter plot are the normalized signal values of the control and sevoflurane groups (log2 scaled). Expression values are represented in different colors, indicating expression levels above and below the median expression level across all samples. The red dots indicate increased-expressed genes, while the green dots indicate decreased-expressed genes of the sevoflurane group compared with the control condition (*p* < 0.05, Figure 2A). Hierarchical cluster analysis showed differentially expressed genes in the sevoflurane group compared to the control condition. The non-paired *t*-test was used to determine the differences between the two groups. We identified 814 differentially expressed genes (DEGs, *p* < 0.05), 631 of which were down-regulated and 183 were up-regulated (Figure 2B).

To explore the pathophysiologic mechanism of sevoflurane anesthesia-related brain dysfunction, enrichment analysis was carried out. The significance of GO and KEGG enrichment was calculated by the hypergeometric distribution and Fisher exact test, and a lower *p*-value [higher −log_10_ (*p*-value)] indicated that the specific term was more significantly enriched. The significance of GO and KEGG enrichment was calculated by the hypergeometric distribution and Fisher exact test, and a lower *p*-value indicated that the specific term was more significantly enriched. The results of DAVID for GO and KEGG analysis revealed that 44 terms of biological process, 16 terms of molecular function, and 18 terms of the cellular component were significantly enriched after sevoflurane anesthesia (*p* < 0.05), respectively. Among them, oxidative stress, metabolism, aging, and neurodegeneration were most enriched biological processes and changed functions after sevoflurane. Six GO terms of oxidative stress, 28 GO and 7 KEGG terms of metabolism, 12 GO terms of aging and neurodegeneration, and 20 terms of the cellular component were significantly enriched.

**Figure 3:**
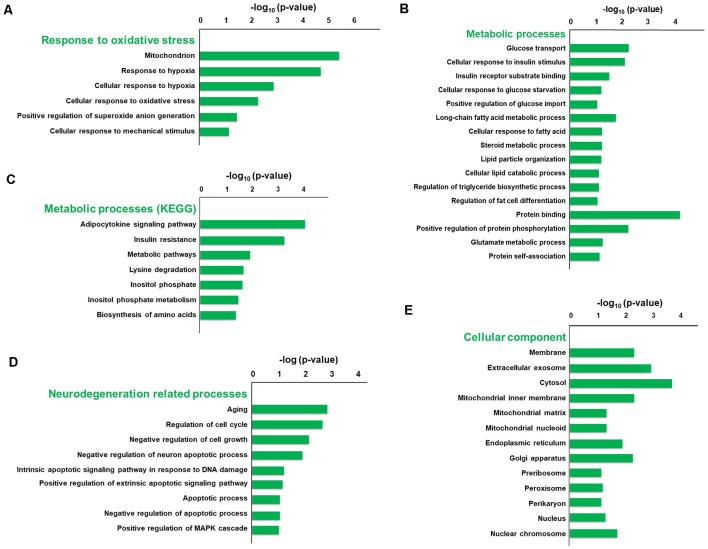
Gene Ontology (GO) and KEGG enrichment analysis of differentially expressed genes using the DAVID database. The typical terms were displayed and ranked according to the value of −log_10_ (*p*-value), including six GO terms related to oxidative stress** (A)**, 28 GO terms and seven KEGG terms related to metabolism **(B,C)**, 12 GO terms related to aging and neurodegeneration **(D)**, and 20 GO terms of cellular component **(E)**.

The typical terms were displayed and ranked according to the value of −log_10_ (Enrichment *p*-value, Figure 3). Previous studies have found that oxidative stress is involved in the development of AD, Parkinson’s disease and other neurodegenerations (Giasson et al., 2000). And we focused firstly on the enriched GO terms related to oxidative stress. The terms include mitochondrion, response to hypoxia, cellular response to hypoxia, cellular response to oxidative stress, positive regulation of superoxide anion generation and cellular response to mechanical stimulus, and the −log_10_ (*p*-value) of these terms were 5.32, 4.6, 2.79, 2.21, 1.37 and 1.13, respectively (Figure 3A). Our previous results also indicate that elevated reactive oxygen species (ROS) and related DNA damage are involved in anesthesia-related pathophysiological changes (Ni et al., 2017).

Enriched metabolic terms including energy, carbohydrate, lipid, nucleotides and amino acid metabolism, were associated with the most potential target genes (Figures 3B,C). Glucose transport, long-chain fatty acid metabolic process, and protein binding were top significant enriched GO terms in carbohydrate, lipid, and amino acid-related metabolic process, and the −log_10_ (*p*-value) were 2.26, 1.75 and 4.18, respectively (Figure 3B). Then KEGG pathway analysis was employed to reveal involved molecular interaction, reaction and relation networks after sevoflurane anesthesia. Figure 3C highlighted seven significantly enriched signaling pathways in our annotation with –log_10_ (*p*-value) > 1, including certain signaling pathways such as adipocytokine signaling pathway, insulin resistance, metabolic pathways, lysine degradation, inositol phosphate metabolism, and biosynthesis of amino acids. Previous studies show that apolipoprotein E plays a central role in the clearance of β-amyloid (Aβ) from the brain (Cramer et al., 2012) and insulin pathway is involved in resistance to oxidative stress and aging (Byrne et al., 2014). The present results indicate that the sevoflurane anesthesia cloud also alters multiple metabolic pathways and processes.

Enriched GO terms related to aging and neurodegeneration include aging, regulation of cell cycle, negative regulation of cell growth, negative regulation of neuron apoptotic process, intrinsic apoptotic signaling pathway in response to DNA damage by p53 class mediator, positive regulation of extrinsic apoptotic signaling pathway *via* death domain receptors, apoptotic process, negative regulation of apoptotic process and positive regulation of MAPK cascade, and −log_10_ (*p*-value) of these terms were 2.84, 2.68, 2.14, 1.87, 1.20, 1.14, 1.06, 1.04 and 1.01, respectively (Figure 3D). Apoptosis and related pathway contribute to the Aβ neurotoxicity in AD, neurodegeneration and dementia (Gervais et al., 1999), and DNA damage also involves in the processes of aging (Lu et al., 2004). And the present results indicate that apoptosis, DNA damage, and other aging neurodegenerative mechanisms are activated, and related gene expressions have been changed in the aged brain after inhaled anesthesia stimulation.

Enriched GO terms of cellular components were ranked according to the location of cellular organelles (from cell membrane to nucleus, Figure 3E). Cytosol, extracellular exosome, membrane, and mitochondrial inner membrane were top significant enriched terms, −log_10_ (*p*-value) of which was 3.9, 3.09, 2.45 and 4.18, respectively. These cellular components could play important roles in sevoflurane-induced pathophysiologic changes. Among which, the mitochondrion is a critical regulator for cell death and mitochondrial dysfunction occurs early and acts causally in multiple disease pathogenesis. Mutations in mitochondrial DNA and oxidative stress both contribute to the aging process, which is the greatest risk factor for neurodegenerative diseases (Lin and Beal, 2006).

Based on GO and KEGG functional annotation and enrichment analysis, involved mechanisms and genes of oxidative stress, metabolism, aging, and neurodegeneration were selected for qPCR verification (*n* = 6 in each group). These included eight DEGs involved in oxidative stress (*Hif2a*, *Hif3a*, *Prkcd*, *Akt*, *Nfe2l2*, *Sod1*, *Scap*, and *Scd2*), six DEGs involved in metabolism (*Scap*, *Hmgcs2*, *Scd2*, *Aldh18a1*, *Glul*, and *Igf1r*), and eight DEGs (*Spdir*, *Ercc4*, *Cdkn1a*, *Hipk2*, *Mal*, *Pmaip1*, *Bmpr1b*, and *Map1lc3b*) involved in aging and neurodegeneration processes. A good consistency between the qPCR and microarray results was confirmed in 17 genes. The non-paired *t*-test was used to determine significant differences between the two groups. However, qPCR validation did not show significant changes for *Hipk2* (103.70 ± 9.061 vs. 100.00 ± 22.04, *p* = 0.8790), *Mal* (115.7 ± 20.74 vs. 100.00 ± 21.49, *p* = 0.6101) and *Bmpr1b* (97.88 ± 17.84 vs. 100 ± 15.52, *p* = 0.9300) after sevoflurane anesthesia. As data quality parameters such as array *p* values and fold change may exert influence on the consistency of the two methods, we assume PCR validations across different experimental conditions are more reliable according to previous studies (Morey et al., 2006). Furthermore, *Hifαs*, *Hmgcss*, and *Cdkn1a* are involved in multiple signaling pathways in oxidative stress, metabolism, and aging/neurodegeneration processes respectively, and were selected for immunofluorescence analysis for their expression levels and regions.

**Figure 4:**
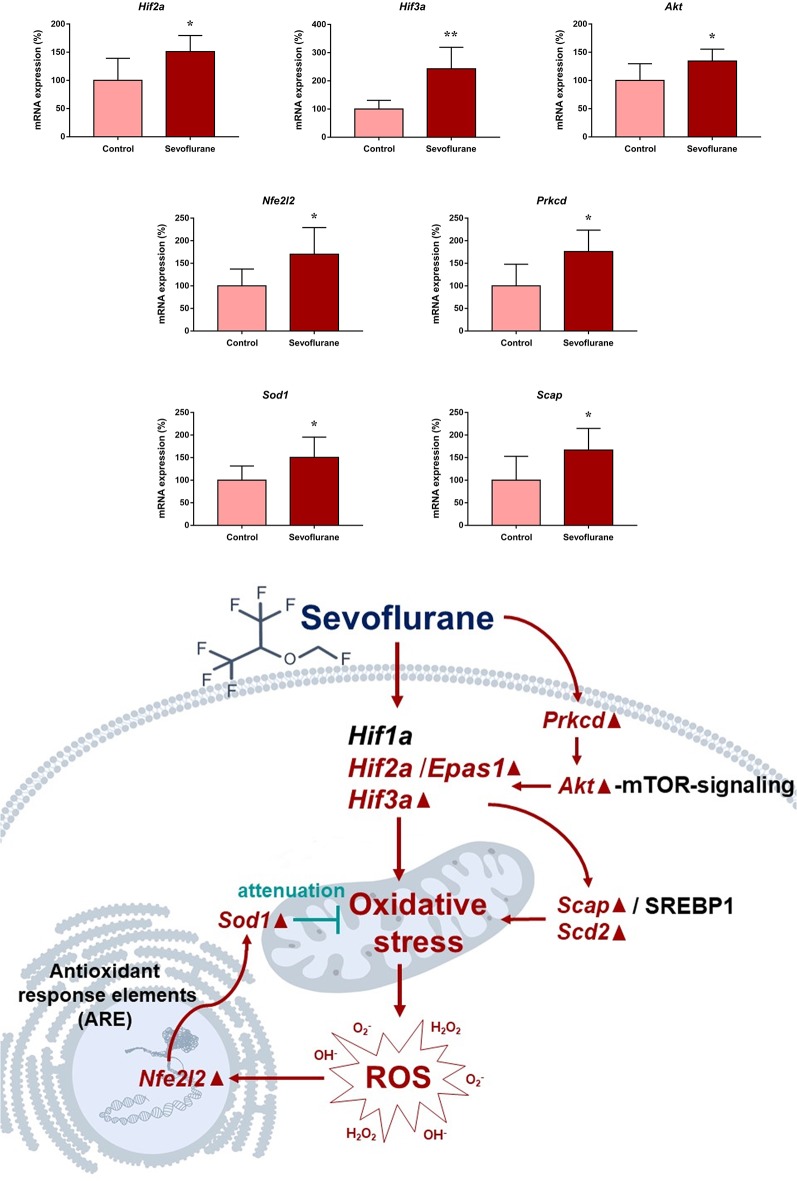
Hypothetical pathway related with oxidative stress identified with altered mRNA expression in sevoflurane group vs. control condition, Regular triangle represents genes up-regulated; inverted triangle represents genes down-regulated; Graphs show the difference in expression of *Hif2a*, *Hif3a*, *Akt*, *Nfe2l2*, *Prkcd*, *Sod1* and *Scap* for sevoflurane anesthesia group and controls (*p < 0.05; **p < 0.01).

**Figure 5:**
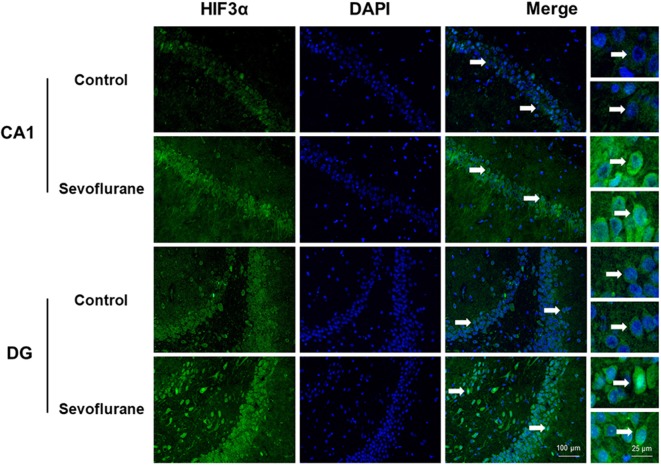
Immunofluorescent staining of hippocampal *hif3a* in the CA1 region and DG region. Immunofluorescence images show RelA (FITC, green) and DAPI (blue) counterstain. In the control condition, RelA was primarily distributed in the cytosol of the pyramidal cell layer, CA1 region and the cytosol of the granular cell layer, DG region. Six hours after exposure, RelA was distributed in both nucleus and cytosol. Arrows point to the typical RelA distribution, which are provided as high magnification images. Magnification 400×, scale bar 100 and 25 μm.

Since close monitoring excluded hypoxia during anesthesia, we assume that sevoflurane may induce perioperative oxidative stress in the brain, and activate related mechanisms and genes. Figure 4 shows oxidative stress-related signaling pathways involved in the aged hippocampus after sevoflurane anesthesia, and differently transcribed genes are shown in red. Sevoflurane activated hypoxia-inducible factors (HIFs) directly, or through *Prkcd* and *Akt*-mTOR-signaling, and DEGs include *Hif2a* and *Hif3a*. Although the increase of *Hif1a* expression was not significant in the present microarray, our previous studies have shown that the expression of HIF-1α increased significantly after inhaled anesthesia (Cao et al., 2018a,b). Activated HIFs involved in *Scap*/SREBP1 and *Scd2* expression increase, and resulted in oxidative stress, then more ROS were generated. It has been reported that the balance of oxygen supply and demand relies on HIFs (Huang, 2013), and *Prkcd* could also control HIFs translation *via* AKT-mTOR signaling under hypoxic conditions (Kim et al., 2016). On another aspect, ROS products activated the *Nfe2l2*-antioxidant response element pathway and increased *Sod1* expression. And *Nfe2l2* mediated *Sod1* increase could attenuate oxidative stress and protect DNA from related damage (Bordoni et al., 2019). Finally, qPCR validation for the mRNA levels of DEGs related to oxidative stress showed significant up-regulation of *Hif2a* (151.29 ± 28.40 vs. 100.00 ± 39.26, *p* = 0.027), *Hif3a* (242.68 ± 76.62 vs. 100.00 ± 30.69, *p* = 0.0017), *Prkcd* (175.87 ± 47.79 vs. 100.00 ± 47.95, *p* = 0.021), *Akt* (134.46 ± 21.08 vs. 100.00 ± 29.77, *p* = 0.043), *Scap* (166.98 ± 47.91 vs. 100.00 ± 53.02, *p* = 0.044), *Scd2* (129.10 ± 20.24 vs. 100.00 ± 19.69, *p* = 0.030), *Nfe2l2* (170.28 ± 59.04 vs. 100.00 ± 37.35, *p* = 0.034) and *Sod1* (8.045 ± 2.39 vs. 5.34 ± 1.689, *p* = 0.0471) after sevoflurane anesthesia compared with control condition. As immunofluorescence shows both the presence and location of protein expression, it was selected for further protein expression verification. Due to previous studies, the hippocampal CA1 region is the substrate for long-lasting potentiation and encoding of synaptic memory (Volianskis and Jensen, 2003), dentate gyrus (DG) serves an important role in engram maintenance and remote memory generalization (Guo et al., 2018). Thus, these regions were selected as the target regions in the present study, and the results showed that the protein expression of HIF-3α in both regions increased after sevoflurane anesthesia (Figure 5).

**Figure 6:**
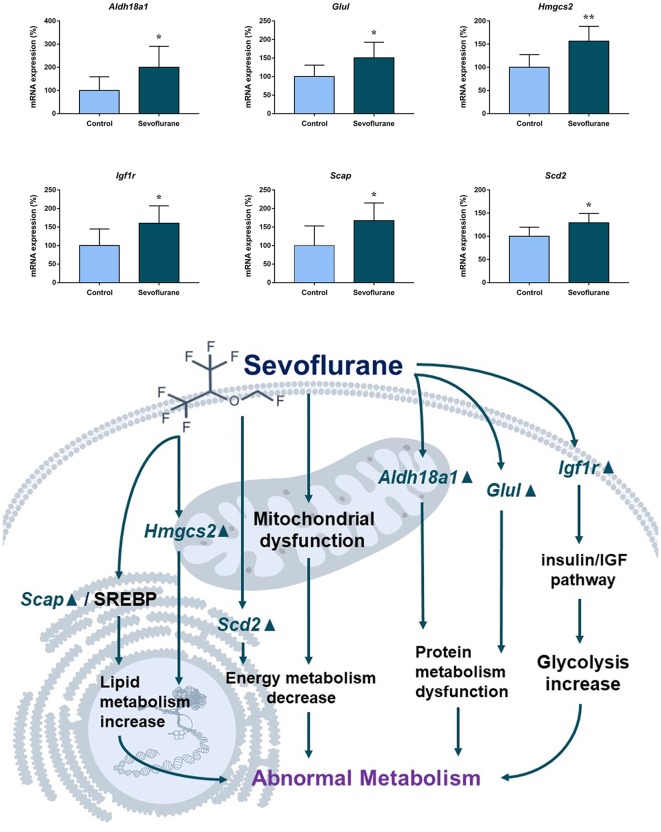
Hypothetical pathway related to metabolism identified with altered DNA expression in the sevoflurane anesthesia group in comparison with the control group. The regular triangle represents genes up-regulated; inverted triangle represents genes down-regulated; Graphs show the difference in expression of *Aldh18a1*, *Glul*, *Hmgcs2*, *Lgf1r*, *Scap*, and *Scd2* were for sevoflurane anesthesia group and controls (*p < 0.05; **p < 0.01).

**Figure 7:**
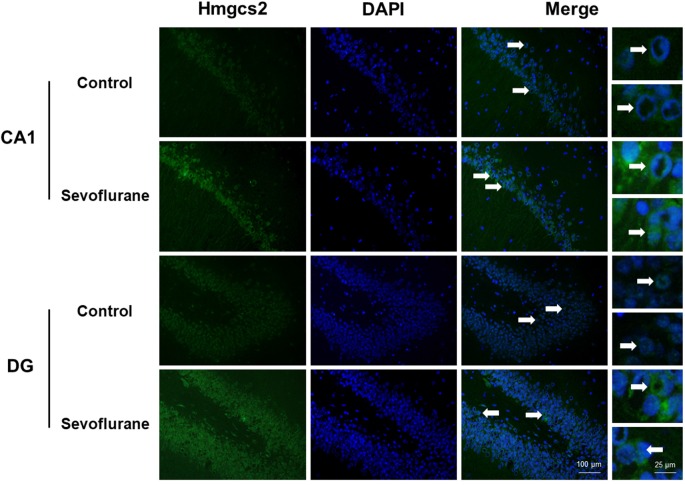
Immunofluorescent staining of hippocampal *Hmgcs2* in the CA1 region and DG region. Immunofluorescence images show RelA (FITC, green) and DAPI (blue) counterstain. In the control condition, the staining intensity of RelA in the cytosol of the pyramidal cell layer, CA1 region and the cytosol of the granular cell layer, DG region are weak. Six hours after exposure, the staining intensity of RelA in the cytosol of cells in both the CA1 region and the DG region is obviously increased. Arrows point to the typical RelA distribution, which are provided as high magnification images. Magnification 400×, scale bar 100 and 25 μm.

Figure 6 shows metabolism-related signaling pathways involved in the aged hippocampus after sevoflurane anesthesia with DEGs. Sevoflurane activated *Scap*/SREBP signaling and *Hmgcs2* expression. *Hmgcs2* expression is both sufficient and necessary to the control of fatty acid oxidation in cells (Vila-Brau et al., 2011), and *Scap*/SREBP signaling also involves the process. Sevoflurane induced *Scd2* expression increases and mitochondrial dysfunction related genes. *Scd2* knockdown increases whole-body energy expenditure (de Moura et al., 2016), and the increased expression of *Scd2* could result in energy metabolism decrease. Sevoflurane also increased *Aldh18a1*, *Glul* and *Igf1r* expression. Hypoxia activated proline biosynthesis *via* upregulation of *Aldh18a1* (Tang et al., 2018), *Glul* is an enzyme that converts glutamate and ammonia to glutamine (Eelen et al., 2018), and *Igf1r* plays a central role in glucose metabolism and regulates lifespan and resistance to oxidative stress as well (Holzenberger et al., 2003). Thus, sevoflurane also affected the metabolism of protein and glucose. Then, qPCR validation for the DEGs related to metabolism showed significant changes for *Scap* (167.13 ± 48.00 vs. 100.00 ± 53.06 *p* = 0.0444), *Hmgcs2* (156.52 ± 32.07 vs. 100.00 ± 27.43, *p* = 0.0083), *Scd2* (129.10 ± 20.24 vs. 100.00 ± 19.69, *p* = 0.030), *Glul* (150.55 ± 42.23 vs. 100.00 ± 30.84, *p* = 0.039), *Aldh18a1* (200.42 ± 90.37 vs. 100.00 ± 59.11, *p* = 0.046) and *Igf1r* (160.26 ± 44.83 vs. 4.678 ± 2.097, *p* = 0.047) after sevoflurane anesthesia compared with control condition. And as shown in Figure 7, the protein expression levels of HMGCS2 increased significantly in the CA1 region and DG of the hippocampus after sevoflurane anesthesia.

**Figure 8:**
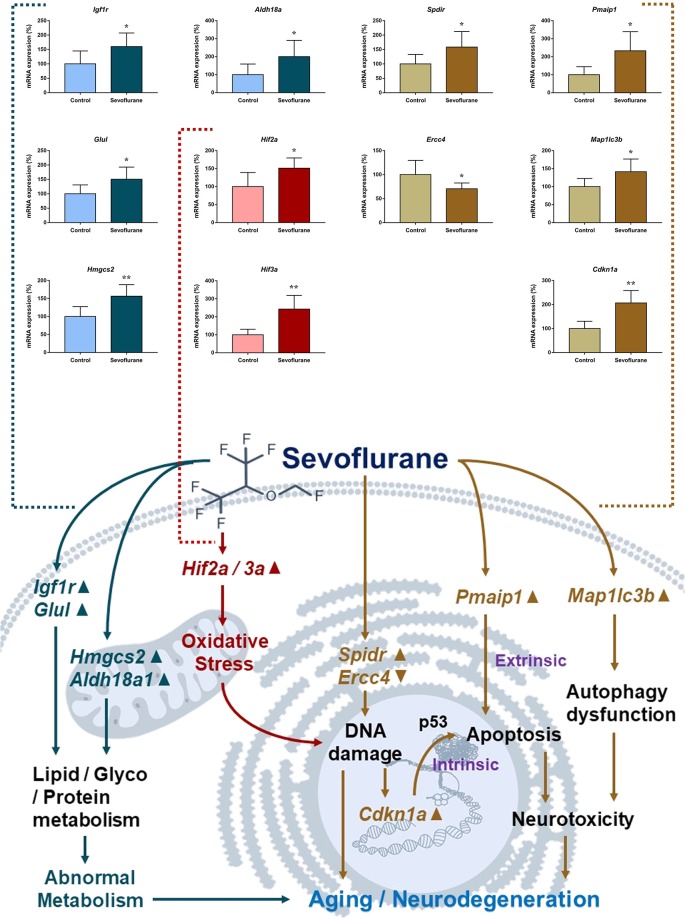
Hypothetical pathway related to metabolism identified with altered DNA expression in the sevoflurane anesthesia group in comparison with the control group. Regular triangle represents genes up-regulated; inverted triangle represents genes down-regulated; graphs show the difference in expression of *Spdir*, *Pmaip1*, *Ercc4*, *Map1lc3b*, and *Cdkn1a* were for sevoflurane anesthesia group and controls (*p < 0.05; **p < 0.01).

**Figure 9:**
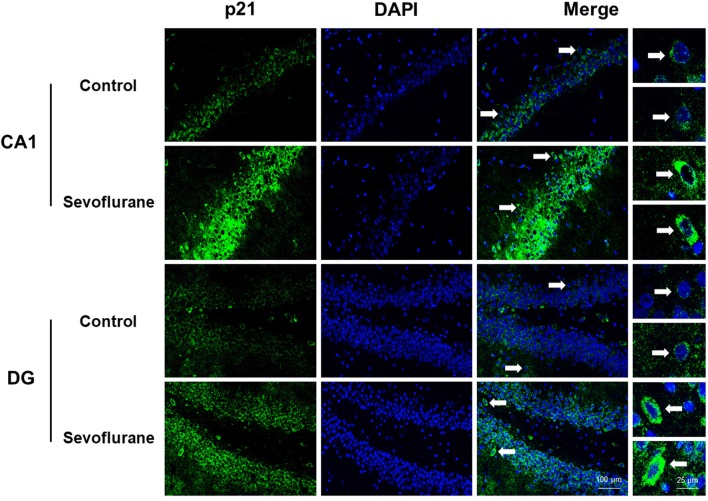
Immunofluorescent staining of hippocampal *Cdkn1a* in the CA1 region and DG region. Immunofluorescence images show RelA (FITC, green) and DAPI (blue) counterstain. In the control condition, the staining intensity of RelA in the cytosol of the pyramidal cell layer, CA1 region and the cytosol of the granular cell layer, DG region are weak. Six hours after exposure, the staining intensity of RelA in the cytosol of cells in both the CA1 region and the DG region is obviously increased. Arrows point to the typical RelA distribution, which are provided as high magnification images. Magnification 400×, scale bar 100 and 25 μm.

Figure 8 shows aging/neurodegeneration related signaling pathways involved in the aged hippocampus after sevoflurane anesthesia with DEGs. Besides oxidative stress and metabolism-related signaling, sevoflurane affected *Spidr* and *Ercc4* expression. *Spidr* involved in DNA repair, and its depletion leads to genome instability and causes hypersensitivity to DNA damaging agents (Wan et al., 2013). *Ercc4* is one of the components of structure-specific endonucleases, which mediate cleavage of DNA structures formed during the repair of collapsed replication forks and double-strand breaks (Svendsen et al., 2009). These changes indicate that sevoflurane could induce DNA damage. DNA damage is a unifying mechanism in neurodegeneration (Ross and Truant, 2017), and could also involve in brain function alteration after anesthesia. Sevoflurane increased *Cdkn1a* and *Pmaip1* expression. Activation of the tumor suppressor p53 by DNA damage induces either cell cycle arrest or apoptotic cell death, and the cytostatic effect of p53 is mediated by transcriptional activation of the cyclin-dependent kinase inhibitor p21 (coded by *Cdkn1a*, Seoane et al., 2002). *Cdkn1a* was also associated with aberrant cell-cycle and apoptosis (Khan et al., 2018), and *Pmaip1* was associated with apoptosis (Zhao et al., 2014). Sevoflurane induced *Map1lc3b* expression, which plays an important role in autophagy (Samdal et al., 2018). Then, qPCR validation for the DEGs related to aging/neurodegeneration showed significant changes for *Spidr* (158.56 ± 53.87 vs. 100.00 ± 33.15, *p* = 0.047), *Ercc4* (70.43 ± 12.22 vs. 100.00 ± 29.81, *p* = 0.049), *Cdkn1a* (206.99 ± 51.71 vs. 100.00 ± 30.31, *p* = 0.0014), *Pmaip1* (232.86 ± 106.46 vs. 100.00 ± 44.78, *p* = 0.018) and *Map1lc3b* (141.64 ± 35.05 vs. 100.00 ± 22.76, *p* = 0.034) after sevoflurane anesthesia compared with control condition. The protein expression levels of p21 correlated with mRNA results and increased significantly in both the CA1 region and DG of the hippocampus after sevoflurane anesthesia (Figure 9).

**Figure 10:**
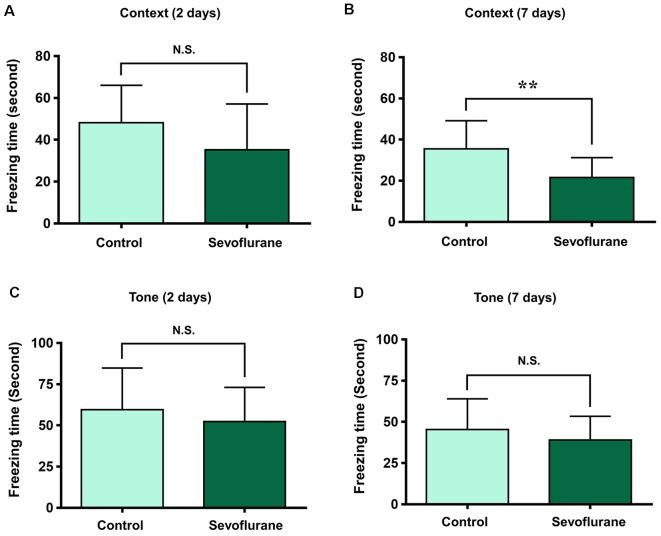
Fear conditioning test (FCT) consisted of a training process at 3 h after anesthesia and evaluations at 2 days and 7 days after anesthesia. The freezing time decreased significantly at 7 days **(B)**, but not 2 days **(A)** after anesthesia in the context test. During the tone test, the freezing time did not decrease significantly at 2 days **(C)** or 7 days **(D)** after anesthesia. **p < 0.01 and N.S.: Not significant.

To assess the relationship between sevoflurane anesthesia and hippocampus-dependent behavioral variations, a subgroup of aged rats was subjected to the FCT, consisted of a training process at 3 h after anesthesia (the same time of genomic expression analysis in the present study), and evaluations at 2 days and 7 days after anesthesia. The results showed that the freezing time decreased significantly at 7 days (21.75 ± 11.32 vs. 36.29 ± 13.50, *p* = 0.0091, Figure 10C), but not 2 days (34.71 ± 19.77 vs. 46.59 ± 20.33, *p* = 0.1609, Figure 10A) after anesthesia in the context test (reflected hippocampus-dependent memory), which suggested that sevoflurane related hippocampal-dependent cognitive dysfunction persisted for a relatively long period. During the tone test, which is related to amygdala function (Li X. Q. et al., 2014), the freezing time did not decrease significantly at 2 days (61.7 ± 31.05 vs. 59.87 ± 26.55, *P* = 0.8777, Figure 10B) or 7 days (45.49 ± 20.75 vs. 42.3 ± 18.19, *P* = 0.6933, Figure 10D) after anesthesia, which suggested that the amygdala function was not grossly impaired.

## Discussion

In the present study, we screened out 814 coding and complex genes that were located across 21 pairs of chromosomes in the aged hippocampus at 3 h after sevoflurane anesthesia, among which, 631 genes were upregulated and 183 genes were downregulated, and the training process of FCT was conducted at the same time. GO and KEGG analysis revealed that 44 terms of biological process, 16 terms of molecular function, and 18 terms of the cellular component were enriched after anesthesia. Among them, oxidative stress (6 GO terms), metabolism (28 GO and 7 KEGG terms), aging and neurodegeneration (12 GO terms) were the most enriched biological processes and changed functions. Candidate genes of oxidative stress (*Hif2a*, *Hif3a*, *Prkcd*, *Akt*, *Nfe2l2*, *Sod1*, *Scap*, and *Scd2*), metabolism (*Scap*, *Hmgcs2*, *Scd2*, *Aldh18a1*, *Glul*, and *Lgf1r*), aging and neurodegeneration (*Spdir*, *Ercc4*, *Cdkn1a*, *Hipk2*, *Mal*, *Pmaip1*, *Bmpr1b*, and *Map1lc3b*) were selected for qPCR verification. A good consistency between the qPCR and microarray results was confirmed, and potential functional genes and signaling pathways were constructed in these biological processes including mitochondria and oxidative stress, metabolism, aging, and neurodegeneration. The FCT results showed that sevoflurane affected memory retrieval at 7 days after anesthesia.

The training process of FCT was performed at the same time of genomic expression analysis (3 h after sevoflurane anesthesia), and the fear conditioning memory retrievals were assessed at 2 and 7 days after the training process (or anesthesia), which both reflected the recent memory. The trend of hippocampus-dependent fear memory decrease was observed at 2 days after anesthesia, but the difference was not significant. While the significant difference of freezing time was observed at 7 days after anesthesia, which indicates that sevoflurane anesthesia could accelerate hippocampus-dependent memory decline. The effects of sevoflurane on memory formation and the hippocampal genomic expression changes during memory formation could be pivotal mechanisms for these effects. Furthermore, the similar phenomenon of contextual fear memory retrieval was observed in both perioperative period (Zhang et al., 2017) and isoproterenol treatment (Qi et al., 2008), and sevoflurane exposure has been reported to affect the level of noradrenaline in the brain (Anzawa et al., 2001).

Mitochondrion and oxidative stress were top enriched terms in cellular component and biological process in the hippocampus after sevoflurane anesthesia based on GO functional annotation, and both control and anesthesia groups received the same concentration of oxygen. Thus, the results indicate that elevated ROS is involved in anesthesia-related pathophysiological changes in the brain. Oxidative stress could be generated as a consequence of antioxidant molecules decrease or inactivation, an increase of ROS and other oxidant molecules, as well as an increase of endogenous metabolites capable of autoxidation (Lushchak, 2014). Oxidative stress is related to lipid droplet accumulation and lipid peroxidation process (Liu et al., 2015), which is involved in the development of neurodegeneration (Sultana et al., 2013). A previous study shows that the effect of intermittent hypoxia on serum triglycerides levels is mediated through HIFs, and HIF inhibitors have a neuroprotective effect in hippocampal apoptosis (Kunimi et al., 2019). HIF-1 impacts on posttranscriptional regulation of SREBP-1 by the increased level of SCAP (Pallottini et al., 2008), which could increase the lipid metabolism and lead to neurodegeneration (Hallett et al., 2019). HIFs also impacts on the of acid-synthesizing enzyme, stearoyl-CoA desaturase (SCD1 and SCD2), which transcription in the hippocampus has been implicated in neurodegeneration (Vozella et al., 2017). The aging retinal pigment epithelium (RPE) expressed higher levels of the Nrf2 (encoded by *Nfe2l2*) target genes compared with the RPE of younger mice under unstressed conditions, suggesting an age-related increase in basal oxidative stress and that Nrf2 signaling is a promising target for novel pharmacologic or genetic therapeutic strategies against aging (Sachdeva et al., 2014).

The results indicate that multiple metabolism-related signaling pathways involved in the process of the aged hippocampus after sevoflurane anesthesia, including energy, lipids, proteins, carbohydrates, etc. Energy metabolism in the aging brain is affected by numerous factors. SCD2 is the main δ9 desaturase expressed in the central nervous system, which has been found playing important role in controlling whole-body energy expenditure (de Moura et al., 2016) and maintaining normal biosynthesis of lipids during early skin and liver development (Miyazaki et al., 2005). SREBP1c is a transcription factor that induces an entire program of de novo lipogenesis primarily in response to increased insulin, and induction of de novo lipogenesis in adipocytes under excess carbohydrate intake is likely to be primarily mediated by SREBP1c as seen in the liver (Kim et al., 1998). SREBP1c is involved in the energy metabolic effects of phenelzine in rats fed a high-fat diet (Mercader et al., 2019). Mass spectrometry and purified protein analysis identified mitochondrial HMG-CoA synthase (HMGCS) as the primary autoantigens, which are ubiquitous and partition with mitochondria, and involved in energy metabolism and oxidative stress (Toivola et al., 2015). HMGCS2 is the regulatory enzyme of ketogenesis in liver mitochondria, which serves as an alternative fuel to reduce the use of glucose during the fasting period, especially in the brain (Nakamura et al., 2014). Ammonia is a toxic product of protein catabolism and involved in glutamate metabolism changes, one of the primary roles of astrocyte is to protect neurons against excitotoxicity by taking up excess ammonia and glutamate and converting it into glutamine *via* the enzyme glutamine synthetase (GLUL). Gene study has found that GLUL is associated with major depressive disorder, and loss of astroglial GLUL is reported in hippocampi of epileptic patients (Zhou et al., 2019). The study also showed that insulin/insulin-like growth factor 1 (IGF1) signaling inhibits age-dependent axon regeneration and involves in neurodegeneration, and growth hormone (GH)/IGF-1 pathway plays a key role in the modulation of the aging process (Byrne et al., 2014).

The sequence of the human genome represents our genetic blueprint, and accumulating evidence suggests that loss of genomic maintenance may causally contribute to aging, and brain aging and neurodegeneration show similarities at the molecular level (Maynard et al., 2015). The most studied molecular pathways involved in neurodegeneration are inflammation and oxidative stress (Fischer and Maier, 2015), metabolism (Citron et al., 2016) and DNA damage (Madabhushi et al., 2014), which are consistent with the pathophysiological process in the hippocampus after sevoflurane anesthesia. The brain consumes oxygen at a relatively high rate, leading to the high exposure of neurons to ROS products. If antioxidants are depleted in the brain, neurons become susceptible to ROS induced DNA damage (Nakae et al., 2000). DNA damage and mitochondrial dysfunction can adversely affect neuronal functions, thus increasing the risk of neurodegenerative disease (Madabhushi et al., 2014). Neurological dysfunction has been found in individuals and mouse models with genetic errors in DNA repair genes (Jeppesen et al., 2011). ERCC4 forms a complex with ERCC1 and is required for the 5′ incision during nucleotide excision repair. And ERCC4 illustrates a critical role in DNA interstrand crosslink repair, and pathogenic variants in this gene cause segmental progeroid syndromes (Mori et al., 2018). In the brains of AD patients and AD mouse models, Aβ plaque-associated Olig2- and NG2-expressing oligodendrocyte progenitor cells, exhibit a senescence-like phenotype through the upregulation of p21 (encoded by *Cdkn1a*) and p16 (Zhang et al., 2019). Autophagy is critical to the maintenance of organismal homeostasis in both physiological and pathological situations. Autophagy protects neurons against regulated cell death by preventing the accumulation of cytotoxic protein aggregates and preserving metabolic homeostasis (Menzies et al., 2015). mTOR inhibitor rapamycin activates autophagy, alleviates the accumulation of Aβ and ameliorates cognitive deficits in mice expressing mutant APP (Caccamo et al., 2010). Combined with the present results, autophagy could also be the therapeutic target for anesthesia-related brain function changes.

Previous studies have shown multiple mechanisms and pathophysiological changes after anesthesia (Ni et al., 2013, 2017; Xie and Xu, 2013; Xie et al., 2013), and genome-wide association studies have been implemented in the studies of brain aging and neurodegenerative diseases (Harold et al., 2009; Lambert et al., 2009; Seshadri et al., 2010). The present study explored sevoflurane anesthesia induced genome-wide changes in the hippocampus of aged rats. Based on functional annotation, mitochondrial dysfunction and oxidative stress, metabolism changes, aging and neurodegeneration, and multiple mechanisms were found to be involved in postoperative pathophysiological processes and function modulations in the hippocampus. Potential genetic regulatory network and involved signaling pathways were established, and genes (include *Hifs*, *Prkcd*, *Nfe2l2*, *Hmgcs2*, *Glul*, *Ercc4*, *Cdkn1a*, *Map1lc3b*, etc.) participate in this genetic regulatory network. These results provide the therapeutic gene targets for brain function modulation and memory formation process, which could be valuable for preventing postoperative brain disorders and diseases, such as PND, from the genetic level in the future.

## Data Availability Statement

The datasets generated for this study can be found in the GEO (submission number: GSE141242).

## Ethics Statement

The animal study was reviewed and approved by Peking University biomedical ethics committee experimental animal ethics branch.

## Author Contributions

YW performed the experiments, analyzed the data, and wrote the original draft of the manuscript. MQ performed the experiments, analyzed the data, and revised the manuscript. YQ and NY contributed to the experiments. KL, JY, and YZ contributed to the data analysis. XG and BM contributed to the manuscript revision. JZ contributed to the experiment design and manuscript revision. CN designed the project, supervised the experiments, drafted and revised the manuscript. All authors read and approved the final manuscript.

## Conflict of Interest

The authors declare that the research was conducted in the absence of any commercial or financial relationships that could be construed as a potential conflict of interest.

## Footnotes

^1^https://david.ncifcrf.gov
